# Predicting NMR Relaxation Using a First-Principles Brownian Dynamics Approach

**DOI:** 10.1021/acs.jctc.5c01827

**Published:** 2026-01-02

**Authors:** Mirco Zerbetto, Sergio Rampino, Antonino Polimeno

**Affiliations:** Dipartimento di Scienze Chimiche, 9308 Università degli Studi di Padova, via Marzolo 1, Padova 35131, Italy

## Abstract

Interpreting time-resolved magnetic resonance experiments, sensitive to slow motions in molecules, requires access to at least the microsecond time scale. Today, all-atom classical molecular dynamics simulations allow exploration of such a long time scale; however, this comes at the price of a considerable computational effort. Stochastic models, based on a hierarchical distinction of the coordinates into relevant (treated explicitly) and irrelevant (treated as generators of fluctuation and dissipation), offer a relatively low-cost solution to this problem. In the past, ad hoc but essentially phenomenological approaches based on Langevin or Fokker–Planck equations have been employed, which are good in catching relevant differences among (even complex) molecular systems, but lack of predictive power since a map between such parameters and atomistic details is not always clear or defined. Recently, a rigorous derivation of a stochastic description of the dynamics of a macromolecule from the complete equations of motion has been provided. In this paper, a computational strategy based on the solution of the Brownian dynamics equations associated with the original model is discussed for the calculation and interpretation of nuclear magnetic resonance relaxation data. The approach merges the ability of stochastic approaches to perform a targeted complexity reduction of the system with the flexibility of molecular dynamics simulations in describing at the atomistic level the time evolution of the system. By expressing the stochastic dynamics in the relevant natural internal coordinates and exploiting the acceleration power of GPU-based hardware, the proposed approach lays the foundations for an effective interpretation of long-time dynamics of generic semiflexible complex molecules.

## Graphical abstract

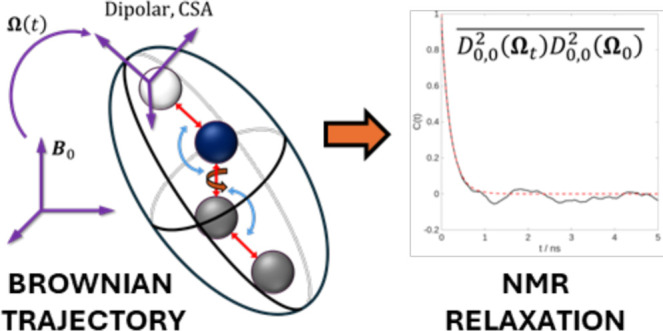

## 1 Introduction

Accessing the long-time behavior of flexible molecules in the liquid phase is important for the interpretation of physical observables sensitive to slow motions, i.e. processes that occur in a time scale from nanoseconds up, such as nuclear magnetic resonance (NMR) relaxation measurements. Classical molecular dynamics (MD) simulations are a tool of choice for describing dynamical phenomena of flexible molecules, and they are usually based on the explicit inclusion of all the system coordinates (all-atom MD simulations). In the context of NMR relaxation, they have first been used as a source of information to parametrize coarse-grained models (for example, to generate conformers of multidomain proteins,^1^ or to access the potential of mean force locally around the probe^2^). Then, as long-time dynamics became accessible, MD trajectories started to be used in the direct calculation of NMR relaxation data, e.g. for small peptides^3^ and proteins, among which ubiquitine,^4^ T4L lysozyme,^5^ GB3.^6^ The usual procedure is to compute long trajectories from 1 to 10 μs (they can be split into multiple calculations of the trajectories of *O*(10^2^) ns) and then separately calculate both the rotational diffusion tensor (for the global tumbling correlation) and proper internal correlation functions. This approach is based on the a priori arbitrary assumption of statistical independence of global and internal motions. Direct calculation of NMR relaxation data without resorting to any a priori assumptions on the correlation function has been performed in small systems, such as the NMR relaxation of the Gd^3+^ complex with water^7^ or in small-medium bulk systems, such as the NMR relaxation of bulk alkanes.^8^ In the former case, reasonable statistics on the dynamics were easily reached because of the very fast motion of the gadolinium–water complex. In the case of the alkanes, statistics was reached by short (2 ns) trajectories since the system is bulk and many replicas of the probe molecule are present in the simulation box. When applying this method to proteins, instead, the simulation box contains just one macromolecule (which is the solute), so that long-time dynamics can be sampled only by controlling the length of the trajectory.

Although the direct simulation of NMR relaxation from MD trajectories is simple in principle, all the coordinates of the system are treated at the same level. This means that the long-time behavior of the molecule must be built up by evolving the whole large set of degrees of freedom, from which eventually the desired slow relaxation processes evolve. This clearly leads to a demanding computational effort since the numerical solution of the Newton equations is usually carried out at most with a 2 fs time step of integration. If the process of interest is characterized by an effective time of the order of a few nanoseconds, then the trajectory must cover at least 100–1000 times that time scale, meaning that simulations in the range of at least the microsecond time scale are required.^4–6^ Such MD runs are today doable but still demanding, especially for large molecules, even on high performance computing hardware.

An interesting viable alternative is to describe the degrees of freedom differently, based on their relevance to the physical observable, by performing a complexity reduction of the problem. When interpreting observations sensitive to long-time dynamics, coordinates can be considered relevant when directly affecting the NMR spectroscopic observable. The remaining coordinates can be considered irrelevant as they have only an indirect influence on the observable, deriving from their coupling to the relevant coordinates. A coarse-grained description, in general, does not describe the latter set of degrees of freedom in detail but attempts to describe their averaged effect on the reduced dynamics of the former set, usually in the form of dissipative and fluctuating forces. This interpretative framework leads to faster and more efficient calculations, but also to a better interpretation of the complex interplay of different relaxation processes. Stochastic modeling approaches based on Langevin/Fokker–Planck formalisms provide a solid framework to perform such a complexity reduction of the molecular system. A well-known phenomenological stochastic approach, introduced to interpret NMR relaxation in proteins, is the model-free method.^9^ In its original form, model-free assumes that the motion of the protein is partitioned in the overall tumbling and a statistically uncorrelated local internal vibration of the probe (e.g., the ^15^N–^1^H bond) described as a sort of wobbling around an equilibrium position. A more refined approach, with an increase in computational complexity, is given by the slowly relaxing local structure model,^2,10^ in which the local dynamics is modeled as a rotator coupled with the global tumbling via some effective potential energy. Model-free can be seen as a limiting case of the slowly relaxing local structure model in the case of large time-scale separation and local symmetric potential. Both approaches are, in essence, phenomenological and require fitting procedures to assign values to the model parameters, thus allowing to describe relevant dynamic features for the observed spectroscopic observable in terms of just a few relevant parameters.

In the past few years, more advanced stochastic models have been proposed which attempt, in specific cases, to pursue descriptions which are phenomenological in nature, and partially based on parameters obtained from short-time MD simulations. For example the diffusive chain model (DCM)^11^ has been introduced, which is based on the selection of a few dihedral angles which are hydrodynamically (that is, through frictional forces) coupled to the global tumbling motion. The parametrization of the model was based on hydrodynamic modeling of the diffusion tensor and the calculation of the potential of the mean force from all-atom MD trajectories. The method has been applied to the interpretation of NMR relaxation data for oligosaccharides,^12–15^ which has proven to be quite effective. However, in order to apply the DCM model, one has to make a selection of the dihedral angles, which can be considered a choice to be checked a posteriori, although the calculation of effective correlation times of the dihedral angles can be shown to be a useful discriminatory parameter.^13^

To put on a more rigorous basis the choices of relevant stochastic variables, a direct derivation of a Fokker–Planck equation to describe the roto-conformation of a flexible molecule has been proposed,^16^ which allows a description of the internal motions in terms of natural coordinates (bond lengths, bond angles, and dihedral angles) and clarifies the coupling between global and internal coordinates. A first treatment based on the hypothesis of local harmonic effective potential, the so-called semi flexible body (SFB) model,^17^ has been introduced, providing semianalytical solutions for small oligosaccharide molecules.^18^ The original model was defined in an inertial (Fokker–Planck) framework, including internal and rotational conjugate momenta. Recently, a further simplifying step has been introduced, assuming a purely diffusive behavior of all coordinates.^19^ In doing so, a new set of internal coordinates have been introduced, as linear combinations of the natural ones, which take the role of generalized “normal modes” with the advantage that the details on the mean force potential and on dissipation are all accounted for in a single tensorial quantity, which provides immediate information on the time scales of the generalized coordinates. Still, the number of degrees of freedom, even for small molecules, is prohibitive for directly solving the predicted dynamics based on a diffusive equation. Employing, instead, Brownian dynamics (BD) simulations can be a way to solve the problem. In the past, BD trajectories to calculate NMR relaxation data have been partially exploited in the context of modeling approaches. A very coarse-grained description was used to calculate the NMR relaxation data of the two-domain protein Pin1.^20^ Dynamics was described in Cartesian coordinates using numerical constraints to fix internal coordinates to be excluded from the modeling. BD trajectories have been used as part of multiscale modeling of lipid membranes,^21^ in parametrization of the stochastic Liouville equation needed because bilayer dynamics occur on time scales comparable to NMR relaxation times. BD trajectories in Cartesian coordinates constrained to match the DCM model have been used to calculate NMR relaxation data for oligosaccharides.^11,13^ A dihedral coordinate BD trajectory, based on the statistical independence of global and internal motions, was used to calculate the NMR relaxation data of ^13^C in alkyl chains linked to an Au nanoparticle.^22^

In this work, we propose an alternative computational approach that takes advantage of the properties of the generalized internal coordinates,^19^ as well as of GPU-based hardware, by calculating the Brownian dynamics (BD) trajectories associated with the original Smoluchowski equation. The idea is to carry out BD trajectory simulations to directly access the correlation functions required to interpret NMR relaxation data, or any kind of time-resolved observables. The usage of BD trajectories in internal coordinates is systematically formalized and analyzed to lay the foundations for an effective approach to long time dynamics of generic semiflexible complex molecules. The description is based on a clear hierarchy of approximations which originates from a full atomistic approach, and gives an efficient modeling tool based on the specific set of generalized coordinates that allows us to analyze a priori the time scales of motion. This information allows us to set the length and resolution of the BD trajectory in advance to ensure the necessary statistics.

In Section II the model is briefly revised and the computational approach is presented, focusing on the numerical strategies adopted to calculate the BD trajectory and to handle it to compute the NMR relaxation data. A brief description of the Brownian Dynamics-NMR (BD-NMR) suite developed in-house is also provided. In Section 3, a sandbox system is used to discuss the properties of the method and to establish how the information on the time scale of motion can be used in setting up the BD simulations. Finally, the calculation of NMR relaxation data for a case-study molecule is reported. In particular, the α-L-Rha*p*-α-(1 → 2)-α-L-Rha*p*-OMe (R2R) disaccharide is considered, since it was studied in the past using MD trajectories, DCM modeling (also in the limit of decoupling between global and internal motion), and the noninertial version of the SFB model.^14,18^ Therefore, results from different modeling strategies can be compared and used to validate the method proposed here, highlighting differences with previous approaches. Our conclusions are laid out in Section IV.

## 2 Theoretical and Computational Approach

### 2.1 Modeling

The stochastic model is based on a full description of the roto-conformational dynamics of the molecule with the solvent treated implicitly as a generator of fluctuation–dissipation and affecting the energetics in a Boltzmann-averaged way. Such an approximation is based on the a priori assumption of solvent coordinates being fast with respect to the solute coordinates to allow one to perform a Zwanzig-Mori projection of the solvent degrees of freedom. This means that no relevant specific solute–solvent interactions are included in the present formulation. Under such an approximation, the projection of the solvent coordinates from the deterministic equations of motion of the whole system described in the set of rigid body + internal natural (Z-matrix) coordinates has been previously discussed in the more general Fokker–Planck form,^16,17^ and in the reduced diffusive limit.^19^ Taking as valid the latter level of approximation and considering the translational symmetry of the problem (isotropic, homogeneous liquid phase), the Smoluchowski equation for the time evolution of the conditional probability density ρ­(**x**, *t*|**x**_0_, *t*_0_) of observing the molecule in configuration **x** at time *t* if it was in **x**_0_ at *t*_0_ is

1 $$\rho \left(x,t|x_{0},t_{0}\right)=-\mathrm{\Gamma ̂}\left(x\right)\rho \left(x,t|x_{0},t_{0}\right)$$

where **x** = (**Ω**, **q**), **Ω** is the set of Euler angles that describe the orientation of a molecule-fixed reference frame (MF) with respect to a laboratory frame (LF), and **q** the set of internal coordinates^19^ (see Figure 1). In eq 1, the Smoluchowski operator takes the form

2 $$\mathrm{\Gamma ̂}\left(x\right)=-{\left[\begin{array}{l} \mathrm{M̂}\\ \partial /\partial q \end{array}\right]}^{T}\rho \left(x\right)D\left[\begin{array}{l} \mathrm{M̂}\\ \partial /\partial q \end{array}\right]\rho {\left(x\right)}^{-1}$$

with **M̂** the infinitesimal rotation operator

3 $$D=\left[\begin{array}{ll} D_{RR} & D_{RS}\\ D_{RS}^{T} & D_{SS} \end{array}\right]$$

the diffusion tensor, with **D**_**RR**_, **D**_**SS**_, and **D**_**RS**_, respectively, the rotational, conformational, and coupling parts of the diffusion tensor, and the superscript T stands for matrix transposition. Finally, ρ­(**x**) is the Boltzmann equilibrium probability density

4 $$\rho \left(x\right)=\frac{1}{8\pi^{2}}\frac{\exp\left[-U\left(q\right)/\left(k_{B}T\right)\right]}{{\left\langle \exp[-U\left(q\right)/\left(k_{B}T\right)\right\rangle }_{q}}$$

where *k*_B_ is the Boltzmann constant, *T* the absolute temperature, ⟨···⟩_**q**_ means integration over the internal coordinates **q**, and the fact that the rotational motion is free was used since the medium does not exert orientation confinement on the molecule, so the energy, *U*(**q**) depends only on the internal coordinates.

**Figure 1:**
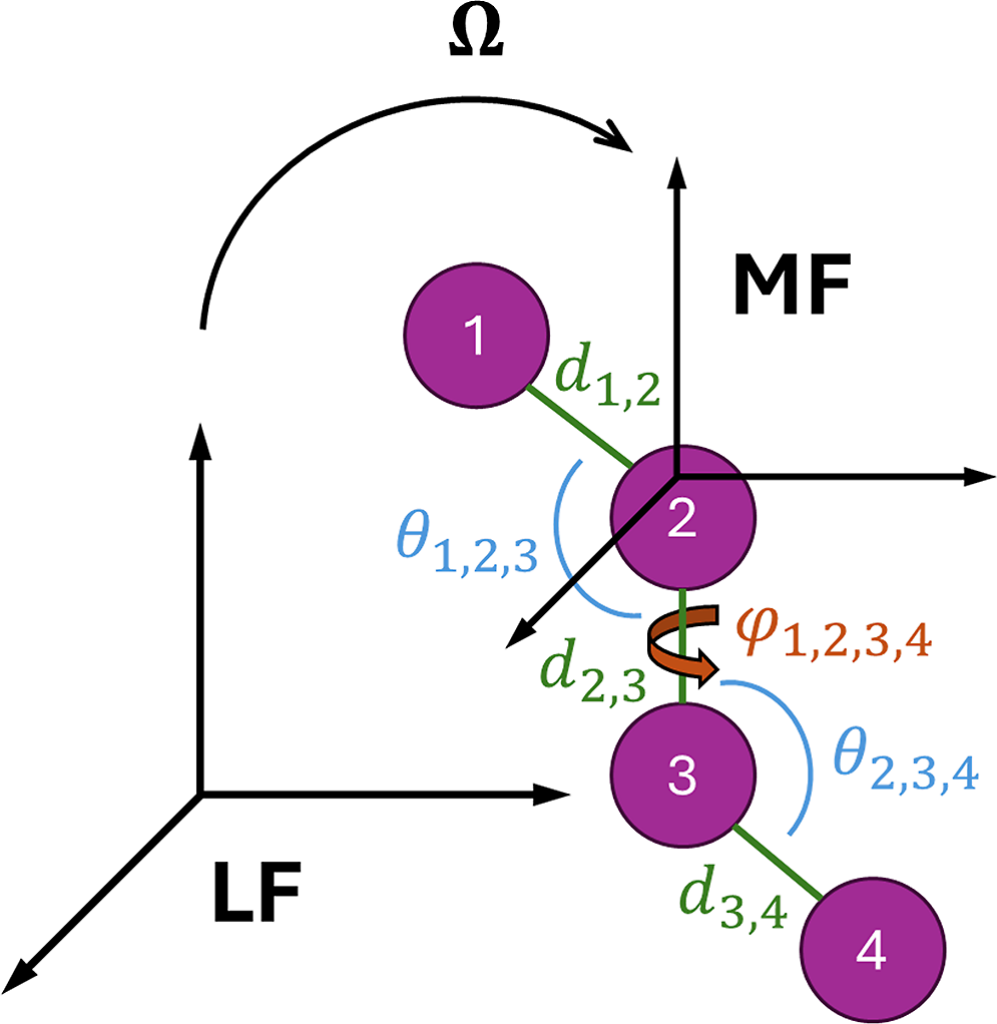
Schematic showing a possible choice of coordinates in the stochastic modeling. The global tumbling is described by the set of Euler angles **Ω**, which give the passive rotation from the laboratory frame (LF) to a molecule-fixed frame (MF). The conformation is described by a set of Z-matrix internal coordinates. In this example, **q** = (*d*_1,2_, *d*_1,3_, θ_1,2,3_, *d*_3,4_, θ_2,3,4_, φ_1,2,3,4_).

We assume the potential to be harmonic, i.e.

5 $$U\left(q\right)\approx \frac{1}{2}{\left(q-q_{0}\right)}^{T}K\left(q-q_{0}\right)$$

with **K** the curvature matrix and **q**_0_ the set of internal coordinates in the minimum energy configuration. Such an approximation may seem too limiting, especially when talking about flexible macromolecules. However, the effect of fluctuations about a single reference structure on the NMR relaxation of the probes is commonly sufficient to interpret experimental data. Basically, this is the implicit modeling of the dynamics in the model-free and in the slowly relaxing local structure approaches. Since NMR relaxation data provides local information, the harmonic model would be inappropriate if activated internal degrees of freedom gave rise to multiple configurations that affected the local geometry and/or the local dissipative properties around the probe. In this case, such alternative relative minimum energy configurations have to be considered if they are close in energy (similar Boltzmann weights) to each other. If this is the case, then, to a first approximation, one can calculate the NMR relaxation data in the different local minima and perform a Boltzmann weighted sum. This idea has been used both in model-free,^1^ and within the slowly relaxing local structure model.^23^ However, such an approach is strictly acceptable if the activation energy is large, e.g. more than 10 *k*_B_ *T*. For smaller barriers, one should consider activated coordinates properly. The DCM model, for example, is implemented to treat such situations.

By modeling the conformational energy as harmonic, it is possible to make the following transformation of coordinates.

6 $$z={\left(k_{B}T\right)}^{-1/2}TK^{1/2}\left(q-q_{0}\right)$$

where **T** is the matrix that diagonalizes **D**_SS_. The internal coordinates **z** are therefore a special set of normal modes (obtained as linear combinations of the Z-matrix coordinates). They all have 0 mean and standard deviation equal to 1. The energetic information is mixed with the dissipative properties, so that the effective time scale of motion is quantified by a single generalized “diffusion” coefficient (one per *z* coordinate) that accounts for all the dynamic effects.

Under this transformation all the internal degrees of freedom are dimensionless, and the diffusion coefficients have the dimension of a frequency, which are the effective frequencies of the motion. As discussed later in the text, this information can be useful for different purposes in the numerical solution of the problem.

Under the transformation in eq 6, and considering that MF is the molecule-fixed frame that diagonalizes **D**_RR_, the rotational and internal parts of the diffusion tensor are diagonal; **D**_RS_ is, in general, a full matrix that provides the entity of coupling between the global tumbling and the conformational coordinates **z**. Finally, the Boltzmann probability density reads^19^

7 $$\rho \left(z\right)=\frac{1}{8\pi^{2}}\frac{\exp\left(-|z{|}^{2}/2\right)}{{\left(2\pi \right)}^{\left(3N-6\right)/2}}$$

where *N* is the number of atoms in the molecule.

### 2.2 Numerical Solution

The target of this modeling is to treat systems with *N* ranging from *O*(10^2^) to *O*(10^4^). Methods based on the representation of the Smoluchowski operator on a proper basis set cannot be employed, since the computational complexity grows exponentially with the number of degrees of freedom. Recently, we proposed and tested a tentative solution approach based on the expansion of the stochastic operator on a set of basis functions.^17,18^ It was based on a projection of the internal coordinates that led to a perturbative expansion directly on the spectral densities, which are the quantities related to the physical observables. However, only the first two terms of such an expansion can be computed efficiently. The limit of this semianalytical method lies in the multiple time-scales of the internal degrees of freedom. The truncation of the expansion requires the **z** coordinates to be fast/moderately fast with respect to the global tumbling of the molecule. This can be expected in small semirigid molecules. In fact, the approach was applicable to a series of oligosaccharides with 100–200 atoms,^18^ but as soon as it was tested on larger molecules, the approximation proved to be inapplicable.

The alternative strategy implemented here is to associate the roto-conformational Smoluchowski equation with a set of Brownian dynamics (BD) equations. The idea is to perform a number of sufficiently long BD trajectories to sample the probability density of the system in time. Moreover, since the transformation in eq 6 is invertible, it is possible to recover the trajectory in Z-matrix, and then in Cartesian coordinates, allowing us to compute any desired observable. With respect to working in Cartesian coordinates, the approach presented here has a number of advantages: *i*) usually when calculating potentials of mean force, atom pair interactions enter as coupling terms among internal coordinates; therefore, computing forces in internal coordinates is simpler; *ii*) the approach allows us to work directly on the set of chosen internal coordinates or with their combinations, for which the calculation of Cartesian forces can be cumbersome and too time-consuming; *iii*) any eventual coupling among internal coordinates can be treated simply; *iv*) if a further projection of the internal coordinates is performed, in this approach the remaining degrees of freedom can be easily treated, while in Cartesian coordinates one should introduce constraints, which have impact on the computational efficiency.

Within the SFB model, the dynamics is simply that of a free rigid rotor hydrodynamically coupled to 3*N* – 6 independent harmonic oscillators. To treat the rotational part, an algorithm based on quaternions has been employed,^24^ which allows one to avoid the numerical instabilities that can arise from the use of Euler angles (e.g., the gimbal lock problem). By calling $Q\left(t\right)={\left[Q_{0}\left(t\right)Q_{1}\left(t\right)Q_{2}\left(t\right)Q_{3}\left(t\right)\right]}^{T}$ the quaternion at time *t* that expresses the orientation of MF with respect to LF, then the discretized BD equations of motion in the Euler scheme are as follows.

8 $$\begin{array}{lll} \left[\begin{array}{l} Q\left(t+\Delta t\right)\\ z\left(t+\Delta t\right) \end{array}\right] & = & -\Delta tW\left(Q\left(t\right)\right)D\left[\begin{array}{l} 0_{3}\\ z\left(t\right) \end{array}\right]+\sqrt{2\Delta t}W\left(Q\left(t\right)\right)D^{1/2}n\left(t\right)+\left[\begin{array}{l} \lambda Q\left(t\right)\\ 0_{N_{S}} \end{array}\right]\\ & & =\left[\begin{array}{l} \mathrm{Q̃}\left(t+\Delta t\right)\\ z\left(t+\Delta t\right) \end{array}\right]+\left[\begin{array}{l} \lambda Q\left(t\right)\\ 0_{N_{S}} \end{array}\right] \end{array}$$

where *N*_S_ = 3*N* – 6, **n**(*t*) is a white noise with 0 mean and variance ⟨*n*_*i*_(*t*)*n*_*j*_(*t*′)⟩ = δ­(*t* – *t*′)­δ_*i*,*j*_, and the matrix **W**, with dimensions (4 + *N*_S_) × (3 + *N*_S_), reads

9 $$W\left(Q\right)=\left[\begin{array}{ll} b\left(Q\right) & 0_{4\times N_{S}}\\ 0_{N_{S}\times 3} & 1_{N_{S}\times N_{S}} \end{array}\right]$$

and

10 $$b\left(Q\right)=\frac{1}{2}\left[\begin{array}{lll} -Q_{1} & -Q_{2} & -Q_{3}\\ Q_{0} & -Q_{3} & Q_{2}\\ Q_{3} & Q_{0} & -Q_{1}\\ -Q_{2} & Q_{1} & Q_{0} \end{array}\right]$$

Finally, in eq 8, a Laplace multiplier is used to ensure normalization of the quaternion. At each time step, λ is obtained by solving for

11 $$\lambda^{2}+2\lambda Q\left(t\right)\cdot \mathrm{Q̃}\left(t+\Delta t\right)+{\mathrm{Q̃}}^{2}\left(t+\Delta t\right)=1$$

The Euler scheme in eq 8 can be optimized by implementing a simple Runge–Kutta scheme,^25^ where a first “virtual” jump is performed following the Euler scheme, and then the actual jump is performed by considering the average force and the average dissipative properties between the starting and the virtual points. Here, it is assumed that the diffusion tensor does not depend on the molecular geometry; therefore, the Runge–Kutta time evolution is

12 $$\begin{array}{lll} \left[\begin{array}{l} Q_{RK}\left(t+\Delta t\right)\\ z_{RK}\left(t+\Delta t\right) \end{array}\right] & = & -\frac{\Delta t}{2}\left\{W\left(Q\left(t\right)\right)D\left[\begin{array}{l} 0_{3}\\ z\left(t\right) \end{array}\right]+W\left(Q_{EUL}\left(t+\Delta t\right)\right)D\left[\begin{array}{l} 0_{3}\\ z_{EUL}\left(t+\Delta t\right) \end{array}\right]\right\}+\\ & & \sqrt{\frac{\Delta t}{2}}\left\{W\left(Q\left(t\right)\right)+W\left(Q_{EUL}\left(t+\Delta t\right)\right)\right\}D^{1/2}n\left(t\right)+\left[\begin{array}{l} \lambda Q\left(t\right)\\ 0_{N_{S}} \end{array}\right] \end{array}$$

where ${\left[Q_{EUL}\left(t+\Delta t\right)z_{EUL}\left(t+\Delta t\right)\right]}^{T}$ is the solution to the Euler expression given in eq 8, while ${\left[Q_{RK}\left(t+\Delta t\right)z_{RK}\left(t+\Delta t\right)\right]}^{T}$ is the solution for the Runge–Kutta scheme. The latter allows one to use a higher time step of integration than the one in the Euler scheme, but requires approximately twice the time to propagate the trajectory with respect to the Euler scheme. Therefore, this Runge–Kutta implementation is advantageous when the time step of integration can be chosen more than twice the one required in the Euler implementation.

### 2.3 Parametrization

To parametrize the SFB model, two quantities are required: the hydrodynamic diffusion tensor (**D**_hydro_) and the matrix of the curvatures (**K**). They are both required to compute the diffusion tensor **D** for the description of the dynamics in the coordinates (**Ω**, **z**).

To compute **D**_hydro_, a reference frame fixed on the molecule is initially defined using three reference atoms, arbitrarily chosen but not collinear. The tensor is then computed using hydrodynamic arguments, using a methodology presented elsewhere.^26^ The interested reader is directed to the cited paper for details, but here we briefly summarize our recipe: the molecule is represented by a number of beads (here, the atoms) that translate into a continuous medium with the constraints given by the bonds. The approach computes the translational friction tensor of the beads using the Stokes–Einstein relation for each bead and the Rotne-Prager model^27^ for the hydrodynamic coupling. Then, a geometric matrix is used to convert the translational Cartesian velocities into the generalized velocities of center of mass translation, global tumbling (angular velocity), and rates of change of the internal Z-matrix coordinates. By projecting the translational motion, the resulting diffusion tensor is represented by a (3 + *N*_S_) × (3 + *N*_S_) matrix, with rotational, internal, and coupling blocks as described above. The parameters of the hydrodynamic model are the temperature, the viscosity of the medium (η), the hydrodynamic radius of the beads (*R*_eff_) and a coefficient (*C*), the value of which depends on whether the boundary conditions are stick or slip. As a first guess, it is assumed that the viscosity is that of the bulk medium, while setting *R*_eff_ = 2 Å and *C* = 6 is a good starting point. Here, it is assumed that all the beads have the same effective hydrodynamic radius. As will be discussed in more detail in the next Section, usually a multiplier of the hydrodynamic diffusion tensor needs to be adjusted. This is the only free parameter of the problem that needs to be tuned when interpreting the experimental data.

The calculation of the matrix of curvatures can be performed in different ways depending on the level at which the solvent should be included. Here, the simplest choice has been taken, i.e. calculating the Hessian energy of the molecule in its minimum-energy conformation in vacuum. To include solvent effects, one may optimize the geometry of the molecule using an implicit solvent approximation, adding a few solvent molecules around specific parts of the molecule, or even by computing the variance-covariance matrix from a short molecular dynamics simulation. In this case, it is important to note that the trajectory should be long enough to let the solvent molecules equilibrate around the solute but short enough to catch the fluctuations of the molecule around the selected minimum energy conformation. If the trajectory is so long that an activated coordinate jumps from a minimum to another one (e.g., a flip in a dihedral angle), then the stochastic dynamics will be nonphysical in the sense that the whole spatial extension of that activated coordinate would be described by a very floppy harmonic potential. Therefore, it is important to pay attention when using molecular dynamics simulations to recover the matrix of curvatures. Under the simplest choice (molecule in vacuum), given a standard force field for the molecule, the Hessian matrix in Cartesian coordinates is readily calculated. Then, it is converted to the internal *N*_S_ × *N*_S_ curvature matrix, **K**.

The diffusion tensor for the dynamics expressed in the shifted coordinates is then calculated as

13 $$D={\left(k_{B}T\right)}^{-1}\left[\begin{array}{ll} E^{T} & 0\\ 0 & T \end{array}\right]{\left[\begin{array}{ll} 1 & 0\\ 0 & K^{1/2} \end{array}\right]}^{tr}D_{hydro}\left[\begin{array}{ll} 1 & 0\\ 0 & K^{1/2} \end{array}\right]\left[\begin{array}{ll} E & 0\\ 0 & T^{T} \end{array}\right]$$

where the dimensions of the identity (**1**) and null (**0**) matrices can be deduced from the dimensions of the other matrices, and **E** is the rotation matrix that diagonalizes the rotational block of the **D**_hydro_ tensor.

Finally, the calculation of the BD trajectory requires selecting the time step of integration (Δ*t*), the total number of trajectory snapshots (*N*_run_), and the lag time between two snapshots (*N*_dump_), i.e., the time resolution of the trajectory. During development and testing of the methodology, it was observed that a trajectory should be at least 1000 times longer than the slower time scale of the motion. In addition, it requires more trajectories to smooth out the correlation functions; a number of 20 trajectories was found to be good enough to fit them with multiexponential functions. Using the Runge–Kutta scheme, it is possible to set the time step of integration equal to half of the fastest time scale. Finally, a resolution such that 10^4^ snapshots are dumped was found to be sufficient to capture important details of the correlation functions in the calculation of NMR relaxation data (see Results section). Given these observations, the parameters of the BD simulation are automatically calculated. Defining **ω** the eigenvalues of **D**, which have the dimensions of frequencies, then the implemented Runge–Kutta integator allows us to set Δ*t* = 1/(2 max­{**ω**}). Also, by default we set *N*_run_ = [(20 × 100/min­{**ω**})/Δ*t*], and *N*_dump_ = *N*_run_/10^4^. Such an automatic configuration of the BD simulation can be used as a starting point. Here, it is stressed that while usually in molecular dynamics simulations the configuration is completely left to the user, in the stochastic SFB model the choice is guided by the eigenvalues of **D** and the fact that one is interested in the long-time dynamics. However, it is important to check the convergence of the calculation, as will be discussed in the next Session.

### 2.4 Implementation

A software suite has been developed designed to calculate NMR relaxation data. It has been named BD-NMR (Brownian Dynamics NMR). It includes both free third-party software, and previously and anew in-house developed codes. Heavy coding has been performed especially to wrap the different software packages and make the calculation flow easy to follow. BD-NMR is delivered on GitHub for free under the GPL-v2.0 license.^28^ In the following paragraphs, a brief description of the workflow is provided, which is also schematized in Figure 2.

**Figure 2:**
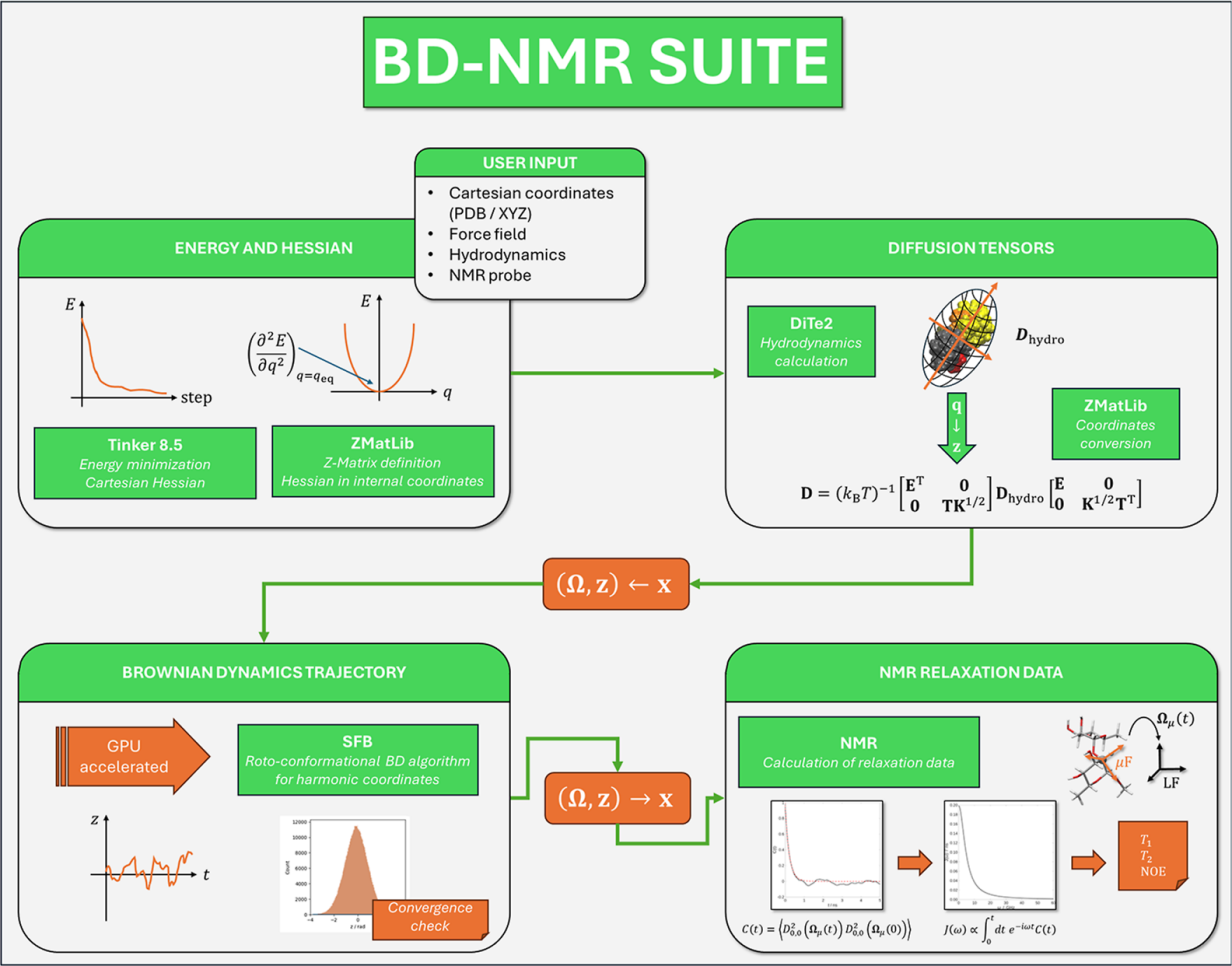
Schematic showing the workflow of data within the BD-NMR suite. The main stages of the calculation are (i) energy minimization and Hessian calculation in internal coordinates provided the initial geometry and the force field, (ii) calculation of the diffusion tensors from user-provided hydrodynamic information and the Hessian, and conversion from Cartesian coordinates (**x**) to rotational (**Ω**) + internal shifted (**z**) coordinates, (iii) calculation of the Brownian dynamics trajectory in (**Ω**, **z**), and conversion to Cartesian coordinates, (iv) calculation of spectral densities of $D_{0,0}^{2}$ functions of the orientation of the dipolar and chemical shift tensors to compute the NMR relaxation data.

#### 2.4.1 Energy Minimization and Hessian Calculation

The SFB model requires that the molecule exists in a representative minimum energy configuration and harmonic fluctuations around such a geometry are allowed. This part is up to the user and constitutes part of the input of the calculation. However, for completeness, BD-NMR is interfaced with the Tinker 8.5 (or higher) software package.^29^ Providing Cartesian coordinates (as PDB or Tinker XYZ files) and a force field, Tinker is used to minimize the geometry and calculate the Hessian in Cartesian coordinates.

A suite of in-house developed tools is used to express the Hessian matrix in internal coordinates. This is handled by the ZMatLib library^26^ which is part of the BD-NMR package and was developed to handle not only the automatic definition of a proper Z-Matrix, but also to provide analytical first and second derivatives of the Cartesian coordinate with respect to the internal coordinates, which are used to transform the Hessian, i.e. the matrix of curvatures; therefore, it corresponds to the matrix **K** appearing in eq 5, which is used for the coordinate transformation **q** → **z**.

#### 2.4.2 Calculation of Diffusion Tensors

The next step is to use the minimum energy configuration and **K** to compute **D**_hydro_ and **D**. The former is estimated using the hydrodynamic approach described above, implemented in the DiTe2 software package.^26^ Parameters for the calculation of **D**_hydro_ are the hydrodynamic radius, the slip or stick boundary conditions, the temperature, and the viscosity.

Then, the square root of **K** is calculated, which is required, together with **D**_hydro_, to compute the matrix **D** following eq 13. The final diffusion matrix is made up of a 3 × 3 diagonal block for rotational motion, a *N*_S_ × *N*_S_ diagonal block for the internal coordinates **z**, and a coupling 3 × *N*_S_ (and its transposed) block.

All matrix operations are implemented on GPU hardware, using the Magma 2.9.0 library^30^ compiled on CUDA linear algebra routines.

#### 2.4.3 Calculation of the BD Trajectory

The Runge–Kutta numerical solution of the Brownian equations of motion for the SFB model, reported in eq 12 has been implemented on GPU. Again, the choice for such an architecture derives from the fact that linear algebra operations are very efficient, especially the Cholesky decomposition required in the calculation of **D** ^1/2^ for the calculation of the random displacement. This is the bottleneck of the calculation of the BD trajectory. However, the Magma library is very efficient for such an operation as it shows a nearly linear scaling up to matrices of dimension 10^4^.^31^

In this step, the eigenvalues of matrix **D**, which correspond to the frequencies of the motions, are used to automatically select the integration time step and the total length of the Brownian trajectory. In particular, if ω_*j*_ is the *j*-th eigenvalue (with *j* = 1, 2, ...,3 + *N*_S_), then the inverse of ω_max_ = max_*j*_{ω_*j*_} is an estimate of the fastest time scale of the dynamics. In a solution scheme based on the discretization of time, the integration time step must respect the inequality Δ*t* ≤ 1/(2 ω_max_), otherwise faster dynamics are not described correctly, and, usually, this implies an instability of the algorithm. In testing the algorithm, it has been found that for the Euler scheme, Δ*t* = 1/(10 ω_max_) is a safe choice. With the Runge–Kutta scheme reported in eq 12, it is possible to increase the time step to Δ*t* = 1/(2 ω_max_). This means that the Runge–Kutta algorithm is roughly 2.5 times faster than the Euler scheme: the time step is 5 times larger, but one Runge–Kutta step takes approximately twice the time of one Euler step. The inverse of the smallest eigenvalue of **D** can instead be considered an estimate of the slowest time scale of the system. The time τ = 1/min_*j*_{ω_*j*_} can be used to guide the selection of the trajectory length. As will be shown in the next Section, a total run time of at least 1000τ is required to have good statistics on all the coordinates of the system.

Finally, the time resolution of the trajectory is also important. For application to NMR relaxation rates, it was found that *O*(10^4^) snapshots provide a good compromise between computation time and convergence.

In summary, BD-NMR is programmed to calculate the eigenvalues of **D**, and automatically sets Δ*t* = 1/(2 ω_max_), to calculate 20 trajectories of length 1000τ, and dump configurations to have a total number of 10 000 snapshots per trajectory (200 000 snapshots in total). The calculation of 20 trajectories has been arbitrarily chosen, and the user is allowed to change everything if the default choices fail.

Once the trajectory in the (**Ω**, **z**) set of coordinates has been calculated, the conversion from **z** to **q** is carried out by inverting eq 6. Finally, ZMatLib is employed to recover the Cartesian XYZ trajectory, which can be used to calculate any observable of the system.

#### 2.4.4 Calculation of NMR Relaxation Data

The package includes a simple C++ code that analyzes the bonding pattern of the atoms in the molecule and recognizes N–H, C–H, and −CH_2_ NMR probes. This information is used to extract the time series of the set of Euler angles, providing the orientation of the dipolar and chemical shift anisotropy (CSA) frames with respect to the laboratory frame. The tilt between the dipolar and CSA frames must be provided as input.

If *A* indicates dipolar or CSA magnetic interactions, the autocorrelation functions

14 $$C_{A,A}\left(t\right)=5\overset{®}{D_{0,0}^{2}\left(\Omega_{A}\left(t\right)\right)D_{0,0}^{2}\left(\Omega_{A}\left(0\right)\right)\;}$$

are calculated, where the overline implies averaging over realizations of the dynamics with the initial condition sampling the equilibrium Boltzmann distribution.

Although the simulation length chosen as discussed above provides sufficient statistics, the tails of the correlation functions will always show some noise. The latter may be removed by increasing by one or 2 orders of magnitude the number of BD trajectories. However, to save time, a multiexponential fitting of the autocorrelation functions is carried out. Work has been done to condition the chi-square so that the fitting proceeds smoothly. If the automatic procedure fails, the user is requested to change the initial guess for the fitting.

Finally, spectral densities are computed, using the Fourier transform of the correlation functions, and used in the evaluation of the NMR relaxation data, following standard equations.^32^

## 3 Results

### 3.1 Sandbox System

It is worth exploring the effect of the properties of the diffusion tensor **D** on the spectral densities. For such a purpose, a sandbox system has been implemented in a Jupyter notebook available to be downloaded.^33^ The purpose is to provide an interested reader with a very simple implementation of the SFB model and its solution via BD trajectories to gain experience before moving on to real molecules. The sandbox system includes the set of three Euler angles for rotation and only one internal coordinate *z*. This implies that the molecule is non linear, with all but one the internal degrees of freedom frozen.

For simplicity, all the frequencies are considered scaled by the isotropic part of the rotational diffusion tensor. The inverse of this frequency is used to scale times. Therefore, all the quantities in this subsection are dimensionless.

To limit the number of parameters, here the rotational part of the hydrodynamic diffusion tensor is considered isotropic. Therefore, because of the above-mentioned scaling, all the elements are set to 1. The conformational hydrodynamic diffusion coefficient, in these units, is set to 10 (internal motions involve fewer atoms than the global tumbling, which is equivalent to an object of smaller area moving in the fluid). For simplicity, only coupling of the internal coordinate with the *D*_*Z*,*Z*_ rotational hydrodynamic coefficient will be considered, ranging from 0 to 1. Finally, the curvature matrix here is a scalar, i.e. the force constant for the harmonic motion of the internal coordinate, *k*. This constant will be expressed in *k*_B_ *T* units and since the internal coordinate has no dimensions, then *k* is also dimensionless. With *c* the hydrodynamic coupling coefficient, the parametric diffusion tensor for this sandbox model reads

15 $$D=\left[\begin{array}{llll} 1 & 0 & 0 & 0\\ 0 & 1 & 0 & 0\\ 0 & 0 & 1 & c\sqrt{k}\\ 0 & 0 & c\sqrt{k} & 10k \end{array}\right]$$

#### 3.1.1 Eigenvalues and Trajectory Control

It is first worthwhile to analyze the 4 eigenvalues of **D** as functions of the two parameters. In this sandbox system, the analytical expressions for the eigenvalues are easy to calculate, since **D** is block-diagonal. They are in ascending order

16 $$\omega_{1}=\left[\left(1+10k\right)-\sqrt{{\left(1-10k\right)}^{2}+4kc^{2}}\right]/2$$

17 $$\omega_{2}=\omega_{3}=1$$

18 $$\omega_{4}=\left[\left(1+10k\right)+\sqrt{{\left(1-10k\right)}^{2}+4kc^{2}}\right]/2$$

The dependence of the first (smaller) and fourth (larger) eigenvalues on both *c* and *k* is reported in Figure 3. When *c* = 0 the first and fourth eigenvalues refer, respectively, to pure rotational and internal motions. With increasing *k*, the time scale separation of the two types of motions increases. This means that the internal energy of the molecule influences the statistical correlation of the global and internal motions. When accounting for hydrodynamic coupling, increasing *c* at fixed *k* causes ω_1_ to decrease and ω_4_ to increase, as global tumbling gets mixed with internal motion. In this case, at given *c* a higher value of *k* is required than in the case *c* = 0 to reach the statistical independence of rotational and internal motions. With such an observation, the tensor **D** may be used to establish a choice between model-free and slowly relaxing local structure, if such phenomenological models are used to interpret NMR relaxation data.

**Figure 3:**
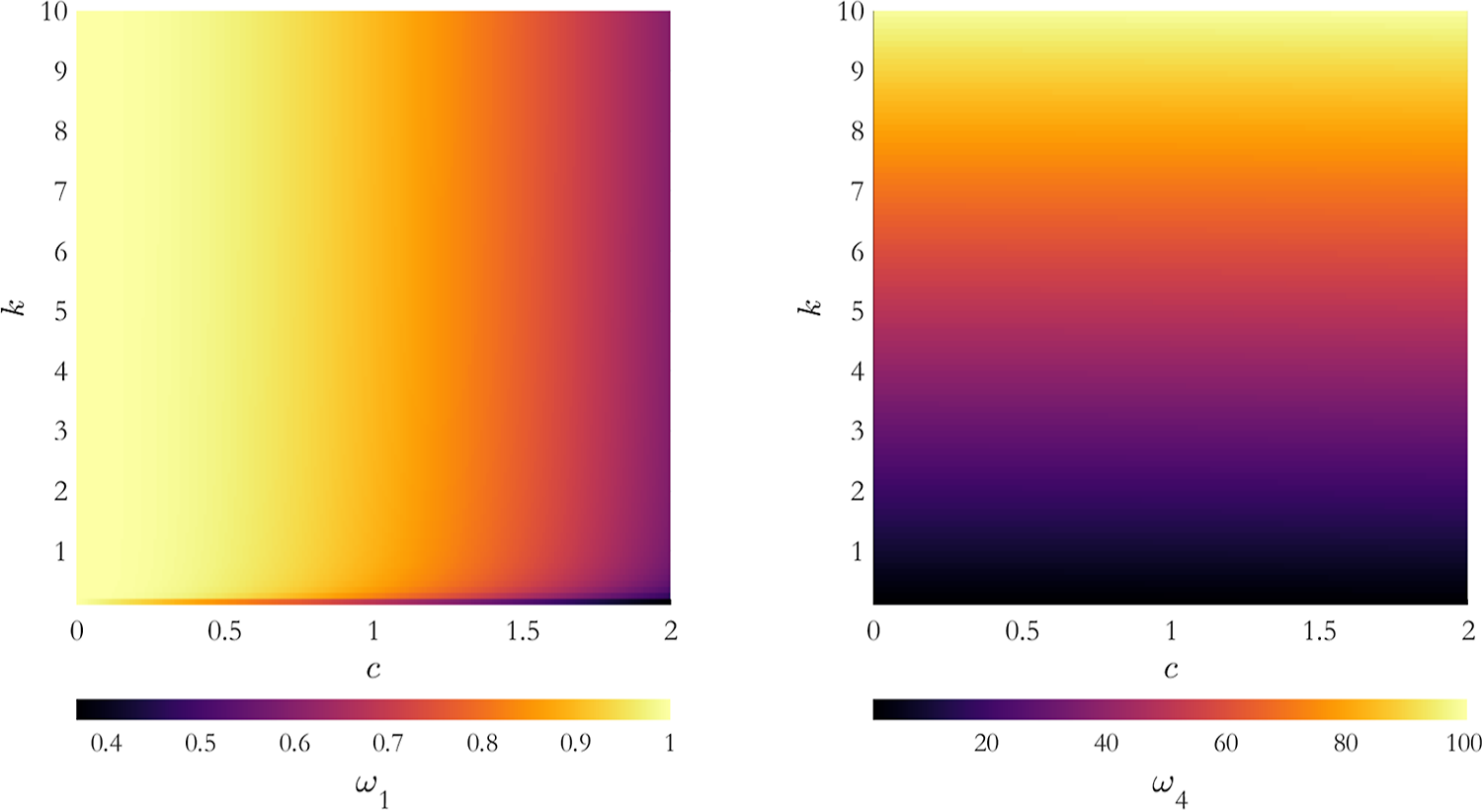
Dependence of the first (left panel), and fourth (right panel) eigenvalues of **D** on both the *c* and *k* parameters. The value of the eigenvalues are represented by a color code.

The eigenvalues of **D** are the frequencies of the dynamics of the system. For this reason, they can be used to determine the time step of integration and the total length of the BD trajectories that need to be calculated. In the scaled units of this example, the time step of integration should be Δ*t* ≤ 1/(2 ω_4_). The dependence of Δ*t* on *k* and *c* is shown in the top panel of Figure 4. As expected, Δ*t* decreases as *k* increases. The effect of the hydrodynamic coupling appears, instead, negligible. For small molecules in water at 298.15 K, the isotropic part of the rotational diffusion tensor is of the order of 10^9^ s^–1^. This means that when *k* is of the order of 10^3^ (expected for rigid parts of the molecule and/or bonds involving hydrogen atoms), one obtains Δ*t* ≤ 100 fs, which is a time step 10 to 100 times larger than that used in MD simulations, which typically is 1 to 2 fs (depending on whether or not the hydrogen atoms are fixed to the heavy atoms). For proteins, the rotational diffusion tensor is one to 2 orders of magnitude smaller, meaning that the motion is “softened out” and a larger time step of integration can be used.

**Figure 4:**
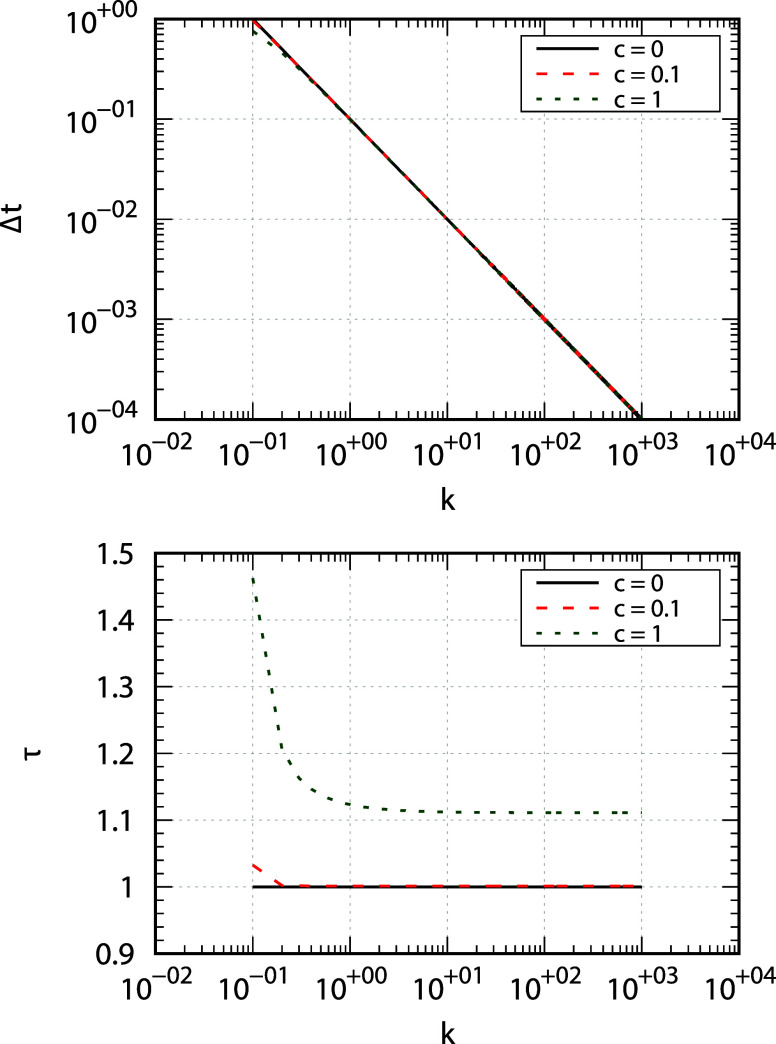
Log–log plots showing the dependence of the (dimensionless) time step of integration Δ*t* (top panel), and the (dimensionless) slowest time τ = 1/ω_1_ (bottom panel) on *k* for *c* = 0 (black, solid line), *c* = 0.1 (red, dashed line), and *c* = 1 (green, dotted line).

Instead, the inverse of the smallest eigenvalue, τ = 1/min_*j*_{ω_*j*_}, provides the slowest time scale of the system. Therefore, the length of the BD trajectory should be decided based on such a quantity. The bottom panel of Figure 4 shows how τ depends on *k* and *c*. The slowest time scale appears to be more sensitive to *c*, the latter determining the limiting value for *k* → ∞. If the hydrodynamic coupling between the global and internal motions increases, the slowest time scale increases, requiring longer BD trajectories to sample the slow motions. In contrast, τ decreases to a plateau value while increasing *k*. Recovering the observation made above, with large *k* the global and internal motions tend to become statistically independent. The plateau may be considered as the condition at which statistical independence may be declared. The bottom panel of Figure 4 shows that (within this diffusive modeling) the plateau is reached at smaller *k* (i.e., for smaller molecules) for a smaller hydrodynamic coupling between rotational and internal motions.

#### 3.1.2 BD Trajectory Convergence

Checking the convergence of the BD trajectory is very simple in the case of the SFB model. Two types of motion are present: the free global tumbling and the harmonic internal dynamics. Rotational dynamics can be conveniently expressed as the three Euler angles **Ω** = (α, β, γ) for the passive transformation of LF to MF. Here, the ZYZ convention is used. Due to the fact that the rotation is free, a trajectory is at convergence if the histograms built from the α­(*t*), cos­(β­(*t*)), and γ­(*t*) time series are uniform distributions in the intervals [−π, π], [−1, 1], and [−π, π], respectively. Instead, each of the internal coordinates *z*, is subject to the same potential *z* ^2^/2. Therefore, the histograms of the *z*(*t*) time series are proportional to the Gaussian exp­(−*z* ^2^/2) with 0 mean and unit standard deviation. The histograms of the time series of the coordinates of the system are useful to check the convergence of the trajectory.

As an example, Figure 5 reports the histogram analysis conducted on BD trajectories of total lengths *t*_tot_ = ατ = α/ω_1_ with α = 1, 10, 100, 1000. As can be seen, while the internal coordinate reaches an acceptable convergence for α = 10, for the global tumbling the trajectory needs to be at least 1000 times longer than the slowest time scale. In fact, this is the main problem in the application of all-atom MD trajectories to interpret NMR relaxation rates: a good sampling of the free rotational motion requires very long trajectories to be calculated. For example, considering a medium protein in water at 298.15 K, the isotropic part of the rotational diffusion tensor is of the order of 10^7^ to 10^8^ s^–1^. If, for simplicity, this corresponds to the slowest time scale, and one is interested in rank 2 rotational correlation functions (see below), then to be at convergence the trajectory should be of the order of 1–10 μs.

**Figure 5:**
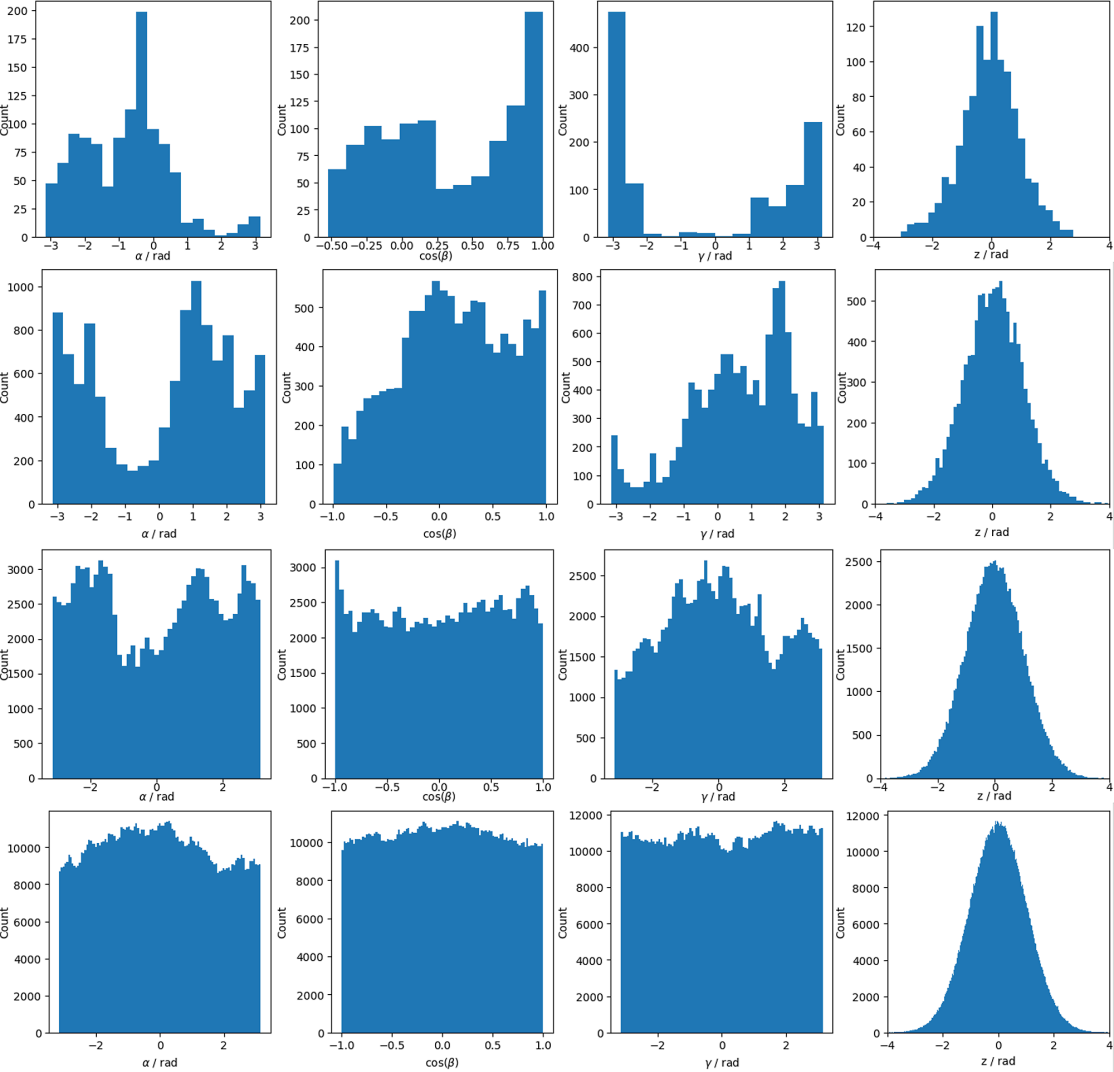
Histograms of α (first column), cos­(β) (second column), γ (third column), and *z* (fourth column) time series for the sandbox system calculated at different trajectory lengths, namely: *t*_tot_ = τ (first row), *t*_tot_ = 10τ (second row), *t*_tot_ = 100τ (third row), and *t*_tot_ = 1000τ (fourth row). Parameters of the calculation are *c* = 1, *k* = 10, Δ*t* = 0.1/ω_4_ (ω_4_ is the largest eigenvalue of **D**).

It is worth noting that one can use the isotropic part of the whole hydrodynamic diffusion tensor as a scaling factor of frequencies (and its inverse for times). The relevance of this observation lies in the fact that the hydrodynamic approach to the diffusion tensor is based on the Stokes–Einstein relation for the friction (ξ) of a sphere translating in a medium

19 ξ=CπRη

where η is the bulk viscosity, *R* is the hydrodynamic radius, and *C* takes two values (4 or 6) based on the boundary conditions (slip or stick). The relation derives from a continuum description of the solvent for a macroscopic solute. The approach adapted to a molecular scale is commonly used, but a scaling factor is usually required to fit the experimental data since the macroscopic factors *C*, *R*, and η cannot be defined a priori in an atomistic description. Past experience allows us to suggest the use of *C* = 6, *R* = 2 Å and bulk viscosity as a good starting point. Then, the scaling factor is the only free parameter of the problem.

A good convergence of the trajectory is very important in interpreting spectroscopic observables sensitive to slow motions, such as NMR relaxation data. In such a context, one has to compute the spectral density of rank 2 Wigner matrices $D_{0,m}^{2}\left(\Omega_{A}\right)$ , where *A* denotes a frame that diagonalizes a magnetic interaction tensor (dipolar, chemical shift anisotropy, quadrupolar, etc.). The correlation function reads

20 $$C_{m,m^{\prime }}\left(t\right)=5\overset{®}{D_{0,m}^{2*}\left(\Omega_{A}\left(t\right)\right)D_{0,m^{\prime }}^{2}\left(\Omega_{A}\left(0\right)\right)\;}$$

where the overline means ensemble average over all realizations of the stochastic process. Figure 6 compares the autocorrelation function *C*_0,0_(*t*) calculated with the BD trajectories obtained with different values of α. It is clear how α = 1000, at least, is needed to catch correctly the details of the curve as well as to have very small noise at long times. This is a very important requirement for interpreting NMR relaxation data, as the long time behavior of *C*_*m*,*m*′_(*t*) influences the 0-frequency value of the spectral density. The latter enters in the expression of the relaxation rates, together with other values of the spectral density at small frequencies (10–100 MHz). If the correlation function is too noisy in the tail, this noise is propagated in the part of the spectral density that is used to calculate the NMR relaxation data.

**Figure 6:**
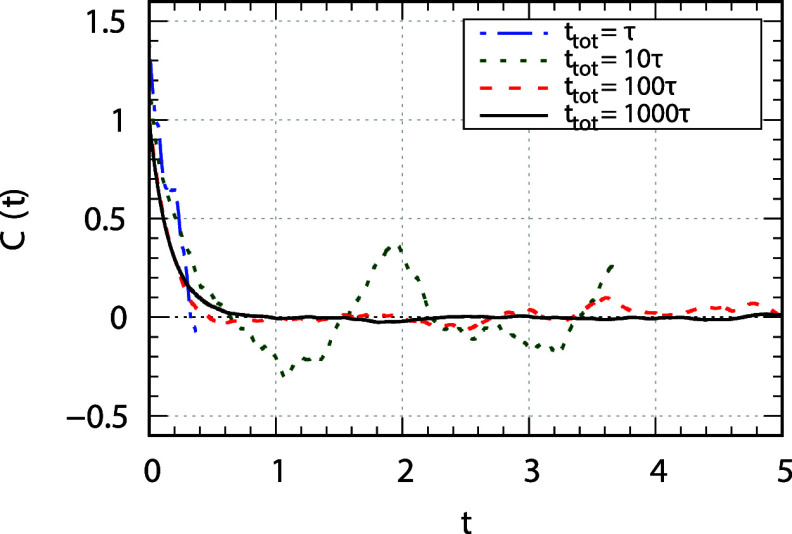
Comparison of the autocorrelation function of the $D_{0,0}^{2}\left(\Omega \right)$ function of the set of Euler angles **Ω** (that provide the orientation of MF with respect to LF) calculated from BD trajectories of lengths: *t*_tot_ = τ (blue, dash-dotted line), *t*_tot_ = 10τ (green, dotted line), *t*_tot_ = 100τ (red, dashed line), and *t*_tot_ = 1000τ (black, solid line). Parameters of the calculation are *c* = 1, *k* = 10, Δ*t* = 0.1/ω_4_ (ω_4_ is the largest eigenvalue of **D**).

### 3.2 Calculation of NMR Relaxation Data

We finally put the developed approach to the test in a real system by calculating NMR relaxation data of α-L-Rha*p*-α-(1 → 2)-α-L-Rha*p*-OMe (R2R, Figure 7) disaccharide in DMSO-*d*_6_ solvent at 298.15 K. As shown in Figure 7, the disaccharide was singly labeled with ^13^C in the C′1 position.^14^ This molecule constitutes a good test system, since interpretation of NMR relaxation data has been attempted in the past using all-atom MD trajectories, the DCM model (including a decoupled version of the model),^14^ and the inertial SFB approach,^18^ allowing us to compare the method presented here with previous approaches.

**Figure 7:**
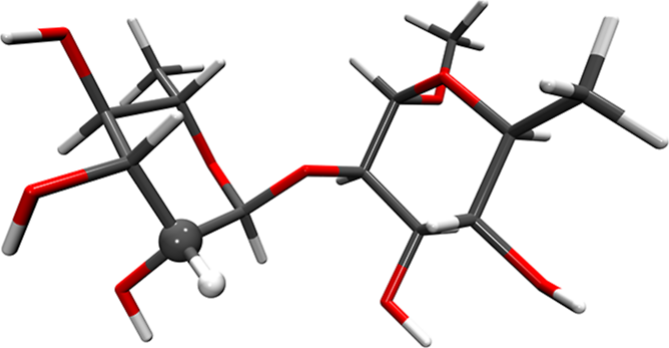
Licorice representation of the R2R disaccharide molecule (C in gray, H in whiter, O in red). The atoms of the ^13^C– ^1^H NMR probe are shown in the ball&stick representation.

The input to BD-NMR consists of an XYZ file in Tinker format and a very short configuration file. Table 1 reports all the information required. Here follows a short description of the keywords. The keyword project is the name of the input XYZ file; refAtoms is a list of four connected atoms, the first 3 atoms determine the starting molecule-fixed frame, while the fourth one can be used to define a specific dihedral angle of interest; ff and grad determine the force field and the tolerance for the energy minimization performed by Tinker; Reff, C, viscosity and temperature are, respectively, the hydrodynamic radius of the atoms, the hydrodynamic boundary conditions, the bulk viscosity and the system temperature, which are used for the calculation of **D**_hydro_; seed is the seed of the generator of pseudorandom numbers, here set to a specific value for reproducibility of the results; nucleus specifies the NMR probe (BD-NMR will automatically search for the possible corresponding probes in the XYZ file); bondLength specifies the length of the C–H or N–H bond; deltaCSA is the anisotropy of the chemical shift tensor; frequency is a list of ^1^H spectrometer Larmor frequencies; calculate specifies the type of NMR relaxation data to be calculated. The hydrodynamic radius reported in Table 1 is the one that provides the best fit results. A comment on this parameter will be made below in discussing the comparison with experimental data. The effective C–H bond length of 1.13 Å takes into account bond librations in the spin Hamiltonian,^34,35^ while the value of δ_CSA_ is set to 0 since it is considered negligible for small molecules with fast tumbling rates. The value of δ_CSA_ and the tilt of the chemical shift anisotropy tensor with respect to the dipolar tensor can be both set in the BD-NMR calculation, but we kept them to 0 as they are commonly neglected for oligosaccharides.^12^

**Table 1:** Configuration Parameters for the BD-NMR Calculation of NMR Relaxation Data of R2R Disaccharide

keyword	values	units
project	r2r	(−)
refAtoms	39 8 15 3	(−)
ff	mm3	(−)
grad	0.01	kcal mol^–1^ Å^–2^
Reff	1.4	Å
C	6.0	(−)
viscosity	2.19 × 10^–3^	Pa s
temperature	298.15	K
seed	123456789	(−)
nucleus	13C1H	(−)
bondLength	1.13	Å
deltaCSA	0	ppm
frequency	600.132 699.973	MHz
calculate	T1 T2 NOE	(−)

The upper panel of Figure 8 reports the eigenvalues of the diffusion tensor. The first thing that stands out is the presence of three eigenvalues that are much separated from the remaining *N*_S_ ones. Such low frequency modes are those that involve the overall motion of all the atoms, i.e. the global tumbling. These three eigenvalues are 5.78 × 10^8^, 6.41 × 10^8^, and 8.62 × 10^8^ s^–1^. If the molecule was completely rigid, the principal values of the hydrodynamic rotational diffusion tensor (calculated with the software package DiTe2 ^26^) would be 5.85 × 10^8^, 6.45 × 10^8^, and 8.76 × 10^8^ s^–1^. The slightly smaller values of the three eigenvalues with respect to the rigid body limit derive from the coupling with internal motions. The other eigenvalues of **D** are at least 2 orders of magnitude larger than the “tumbling” frequencies (quotation marks here are used to remind us that they refer mainly, but not purely, to rotation).

**Figure 8:**
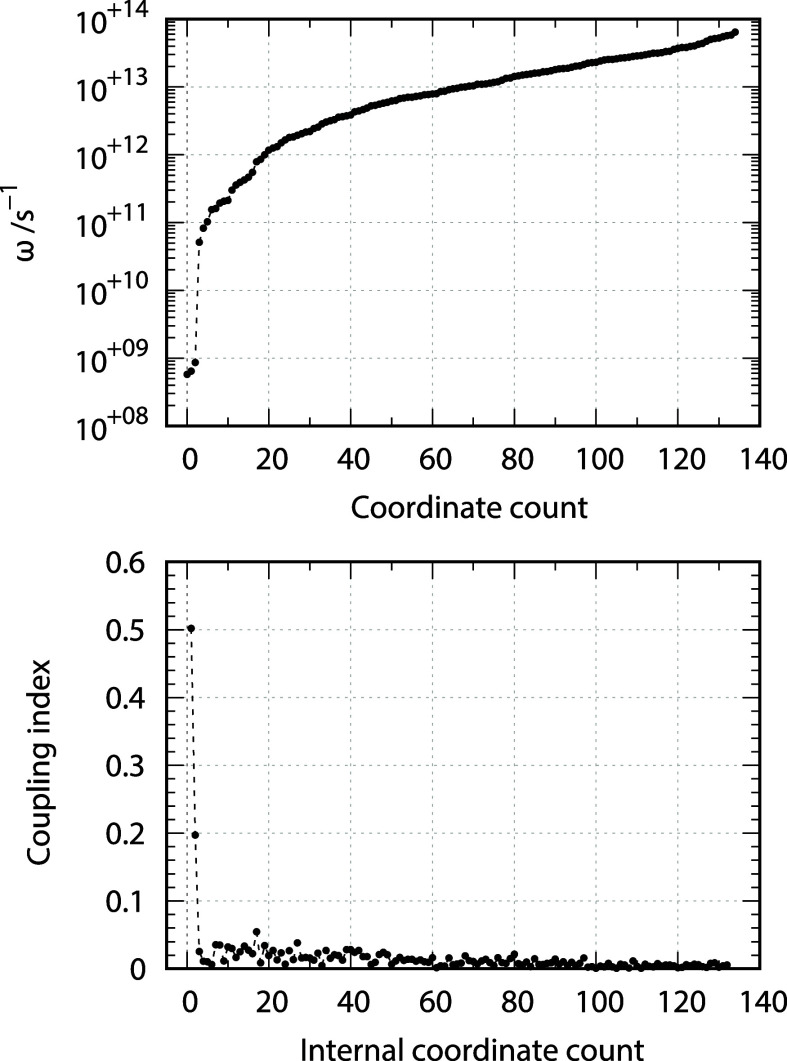
Upper panel: logarithmic plot on the eigenvalues of **D**. Lower panel: coupling index of the *z* coordinates to the rotational motion.

However, such a separation in time scales is not sufficient to call for statistical independence of the global and internal motions. In fact, the lower panel of Figure 8 reports that the first two slowest coordinates *z* are coupled to rotational motion. A collective coupling index CI_*j*_ for the coordinate *z*_*j*_ with rotational motion can be defined as

21 $${CI}_{j}=\sum_{i=1}^{3}\frac{T_{i,j}}{\sum_{j=1}^{N_{S}}T_{i,j}}$$

with

22 $$T_{i,j}=\left|\frac{D_{i,j+3}}{D_{i,i}-D_{j+3,j+3}}\right|$$

It is quite interesting that only two internal coordinates are coupled to the rotational motion. This observation may be used in a process of complexity reduction of the model, which will be important in the treatment of larger systems (e.g., proteins). A more detailed comment on this aspect is reported in the Conclusions. The smallest eigenvalue is used to estimate the longest rank 2 rotational correlation time, τ = 1/(6 × 5.78 × 10^8^) = 0.288 ns. Based on what was observed in the sandbox system, the total length of the trajectory should be set (at least) at 1000τ = 288 ns. The highest eigenvalue of **D** is 6.4502 × 10^13^ s^–1^. Therefore, within the Runge–Kutta scheme, BD-NMR automatically sets the integration time step to half of the inverse of the highest eigenvalue, i.e., Δ*t* = 7.75175 fs. The number of BD steps is adjusted so that the final trajectory can be split into 20 trajectories of the same length. Finally, the dumping rate is set to ensure at least 10^4^ snapshots. All of these operations are automatically handled by BD-NMR. In this example, the total number of snapshots has been estimated to be 4.4 × 10^7^ and the configuration was dumped every 22 steps (a time resolution of 170.5 fs).

As the BD trajectory is computed and converted to Cartesian coordinates, a tool in the BD-NMR suite automatically searches all the C–H bonds that are plausible NMR probes, storing the indexes of the H and C atoms, as well as those of the next atom (X) bonded to C. From the trajectory, the coordinates of these three atoms are used to build the dipolar tensor with the *Z* axis oriented along the C–H bond, the *Y* axis perpendicular to the plane formed by the H–C–X atoms, and the *X* axis determined by the right-hand rule. The orientation of *X* and *Y* axes is arbitrary due to the cylindrical symmetry of the dipolar interaction. Given these definitions, it is possible to compute the time series of the set of Euler angles Ω_dip_(*t*) (where the subscript dip stands for dipolar), which give the orientation of the dipolar frame (the frame where the dipolar interaction is diagonal) with respect to LF. This is the only relevant magnetic tensor in the ^13^C NMR relaxation in this system, since chemical shift anisotropy is negligible.

From the time series of the Euler angles, it is possible to calculate the autocorrelation function of the Wigner function $D_{0,0}^{2}\left(\Omega_{dip}\right)$ , which is the most important contribution to NMR relaxation.^32^ The top panel of Figure 9 reports in a black solid line the normalized autocorrelation function calculated directly from the BD trajectory. Despite the fact that 20 trajectories have been calculated each at least 1000 times longer than τ, the tail of the correlation function is still too noisy to calculate the spectral density. BD-NMR implements an automatic multiexponential fitting procedure (by default, 4 exponential functions are used) to interpolate the correlation function. The fitted correlation function is shown as red dashed line,/in the top panel of Figure 9. Finally, the spectral density of this multiexponential decay is computed. The bottom panel of Figure 9 reports the spectral density, *J*(ω), which is then used to calculate NMR relaxation data.

**Figure 9:**
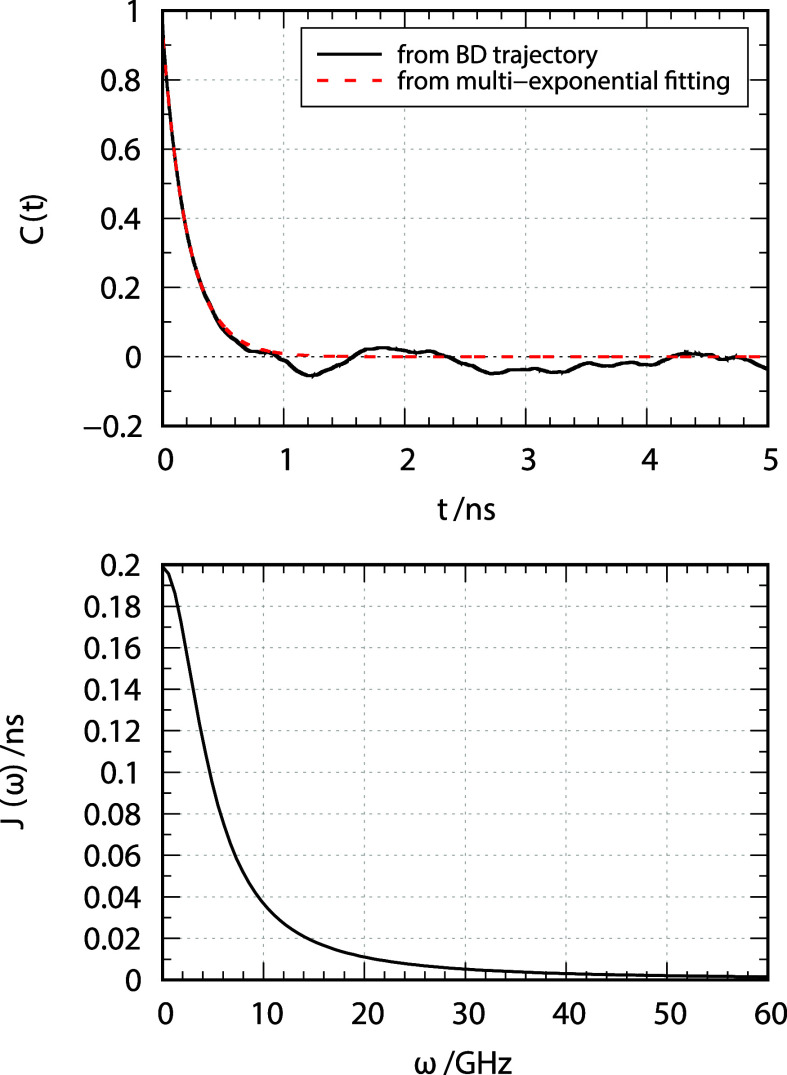
Upper panel: autocorrelation function of the $D_{0,0}^{2}\left(\Omega_{dip}\right)$ function directly calculated from the BD trajectory (black, solid line) and its multiexponential fitting (red, dashed line). Lower panel: spectral density for the dipolar interaction obtained from the Fourier transform of the multiexponential fit of the autocorrelation function.

It is worth inspecting the result of the multiexponential fitting of the dipolar autocorrelation function. By default, 4 exponential terms are used for the fitting, as it was observed that usually this number was sufficient to catch the relevant features of the correlation function. The user can select to use up to 5 exponential terms. The time constants and their weights obtained by the fitting are reported in Table 2.

**Table 2:** Time Constants and Weights of the Exponential Fitting of the Dipolar Autocorrelation Function Shown in Figure 6

exponential #	time constant/ns	frequency/s^–1^	weight %
1	0.218	4.59 × 10^9^	87.5
2	0.113	8.85 × 10^9^	6.9
3	4.88 × 10^–3^	2.05 × 10^11^	2.8
4	2.54 × 10^–6^	3.94 × 10^14^	2.8

Interpreting the tumbling as axially symmetric, with the tumbling diffusion constants reported above, one can calculate the three rank-2 correlation times: ω_0_ = 3.66 × 10^9^ s^–1^, ω_1_ = 3.91 × 10^9^ s^–1^, and ω_2_ = 4.67 × 10^9^ s^–1^. The first two eigenvalues associated to the internal motions, which are the ones with a higher coupling to the global tumbling (lower panel of Figure 8), read 5.13 × 10^10^ s^–1^, and 8.27 × 10^10^ s^–1^. The other eigenvalues range from 10^11^ s^–1^ to 10^13^ s^–1^. From Table 2, the first, most important contribution, is the long-time behavior of the correlation function, which is characterized by a time constant larger than the average rotational frequencies, as an effect of the coupling with the first two harmonic modes. The second exponential is in the order of the slowest harmonic modes (the ones that also happen to be more coupled to the global tumbling). The third and fourth exponential terms have a small weight, still they are required to correctly reproduce the first decay of the autocorrelation function. They count the overall contribution of the very fast librational motions. This poses the attention that not only the correlation functions are multiexponential decays, but also that, in general, the time constants emerge from the coupling between global tumbling and internal motions, as it as also been recently modeled and observed (and validated with molecular dynamics simulations) by modeling NMR relaxation rates of Gd­(III) complexes.^36^

As noted above, the only truly free parameter of the approach is translational friction ξ = π*CR*η. In the past, the use of *C* = 6 and *R* = 2.0 Å usually provided a good starting point. However, working with the SFB model, it was observed that a smaller value of the hydrodynamic radius (or the product *RC*) is required.^18^ Table 3 shows the agreement with the experimental data as a function of the hydrodynamic radius.

**Table 3:** Comparison among Experimental and Calculated NMR Relaxation Parameters as Function of the Hydrodynamic Radius

	600 MHz			700 MHz		
	*T*_1_/ms	*T*_2_/ms	NOE	*T*_1_/ms	*T*_2_/ms	NOE
Exp	440.0	402.6	2.361	475.6	432.9	2.215
*R* = 2.0 Å	379.7	366.8	2.013	422.0	366.8	1.882
% err	–14	–9	–15	–11	–15	–15
*R* = 1.8 Å	394.3	349.3	1.998	438.1	380.0	1.872
% err	–10	–13	–15	–8	–12	–15
*R* = 1.6 Å	415.5	380.7	2.256	456.9	411.8	2.120
% err	–6	–5	–4	–4	–5	–4
*R* = 1.4 Å	440.0	407.7	2.357	480.3	438.6	2.228
% err	0	1	0	1	1	1

Using the hydrodynamic radius of 2.0 Å which provided very good agreement with experimental data within the DCM model,^14^ gives unsatisfactory results with the SFB model. The relaxation rates of *T*_1_ and *T*_2_ are observed to be smaller. This is related to a higher efficiency in thermal relaxation of the magnetization, i.e. a more efficient relaxation of the orientation of the C– H probe due to the presence of, and the coupling to, the internal motions that open new pathways to relaxation. Since they fall into the fast motion regime (correlation times much smaller than the inverse of the Larmor frequency), to increase *T*_1_ and *T*_2_ one has to decrease the effective correlation time, which can be done by reducing the effective radius. In other words, previous models, like DCM, were missing a number of spin–lattice and spin–spin relaxation pathways, so that one had to increase friction to use part of the dynamics in the slow regime to compensate for the lack of these pathways. This reasoning explains why a smaller effective radius of 1.4 Å provides the best fit agreement, with an absolute percentage error ≤1% for all the NMR relaxation data, and a mean percentage absolute error (MAPE) of 0.7%.

A convergence study as a function of the length and the number of the trajectories has been carried out to check the automatic setup of the BD simulation implemented in BD-NMR. In particular, the MAPE has been chosen to measure the overall goodness of fit of the calculated data. Results are reported in Table 4.

**Table 4:** MAPE as Function of the Length and Number of BD Trajectories Calculated with the Best Fit Hydroydnamic Radius of 1.4 Å

	no of trajectories				
length/ns	1	2	4	8	16
1.7	41	21	13	23	18
8.5	6	12	13	8	4
17.0	14	6	2	2	1

It can be observed that to converge the MAPE, at least 16 × 17.0 ns trajectories are needed, corresponding to a total of 277 ns of trajectory, which is close to the time estimated based on the isotropic global tumbling rank 2 rotational correlation time. Table 4 shows that the convergence can also be obtained with shorter trajectories, but increasing their number. For simplicity, when using the automatic setup of the trajectory in BD-NMR, a single long trajectory is computed, which is then split in 20 parts. However, once the eigenvalues of the diffusion tensor are known, the user is free to split the calculation into multiple trajectories (each run must use a different seed for the pseudorandom numbers generator), then merge them and make BD-NMR continue with the calculation of NMR relaxations data.

Table 5 compares the BD-NMR results with those obtained in the past using different modeling approaches, as well as MD simulations. In the table, SALEM refers to calculations carried out in the inertial regime using a perturbative scheme,^18^ DCM refers to calculations carried out with the diffusive chain model where only the ψ dihedral angle is considered as internal coordinate,^14^ DCM dec is a modification of the DCM model where global tumbling and the internal motion have been considered statistically decoupled.^14^ In the DCM model the potential of mean force acting on ψ was obtained from a molecular dynamics trajectory. Finally, MD results have been obtained from a 100 ns trajectory.^14^

**Table 5:** Comparison Among NMR Relaxation Data of R2R Calculated with Different Approaches^a^

frequency	data	Exp.	BD-NMR	SALEM	DCM	DCM dec.	MD
	*T*_1_/ms	440.0	440.0	449.9	429.5	316.4	487.1
600 MHz	*T*_2_/ms	402.6	407.7	420.5	396.0	302.2	452.4
	NOE	2.361	2.357	2.215	2.314	2.620	2.381
	*T*_1_/ms	475.6	480.3	497.5	469.5	335.7	525.1
700 MHz	*T*_2_/ms	432.9	438.6	456.1	426.4	317.5	481.8
	NOE	2.215	2.228	2.150	2.188	2.536	2.290

a For the stochastic models, the results obtained with the best *R*_eff_ are reported. In particular, 1.4 Å for BD-NMR, 1.6 Å for SALEM,^18^ 2.0 Å for DCM and DCM decoupled modeling.^14^ MD results are based on 100 ns-long trajectory.^14^ parameters as function of the hydrodynamic radius.

It can be observed that the BD-NMR calculations compare well with the other stochastic models. In this case, they also show the best agreement with the experimental data. This is due to the fact that the BD-NMR approach includes and treats exactly also degrees of freedom falling in an intermediate time scale, whereas in the DCM modeling these are not present, and the SALEM solution (which includes all the degrees of freedom) is based on a perturbative approximation. Overall, the BD-NMR computation required 2.5 h on a H100 nVidia GPU. The SALEM calculation is much faster (a few minutes), but it is limited to small molecules. The DCM calculation also takes a few minutes, but the procedure of selecting the degrees of freedom to be included in the model still requires an a posteriori check, reducing the predictive power. However, the fact that the DCM model still provided satisfactory agreement with the experimental data suggests that a complexity reduction procedure is desirable for the BD-NMR approach. This is work planned for the next future, especially for the application to macromolecules.

Table 5 also shows the importance of the coupling between internal and global motions. The column “DCM dec.” (which stands for DCM decoupled) shows how bad the results are, all else being equal, if the internal motion is decoupled from the global tumbling. This is a first important point in favor of the BD-NMR with respect to MD simulations. In fact, as described in the Introduction, the MD-based approach is usually built on a factorization of the global and internal correlation functions. However, such an approximation must be justified, and the analysis of the diffusion tensor, **D**, in the shifted coordinates is the quantity to be used for such a purpose. The second advantage with respect to MD simulations is evident by inspecting the last column of Table 5. The MD simulation was 100 ns long, which (as observed above) is a time that is shorter than the minimum length of 288 ns estimated by BD-NMR. This means that, likely, the slowest motions (rotational) are not well sampled. As it was shown in Table 2, the length of the trajectory was not optimal and it was not analyzed by splitting in more parts, which probably would have allowed for a better statistics. While today a sufficiently long MD trajectory can be afforded, the objective of this work is to show that with appropriate stochastic modeling the same information can be recovered with much less computational effort.

In conclusion, BD-NMR merges the level of detail of the dynamics of the molecule that can be achieved from MD simulations with the interpretative capabilities of stochastic methods to apply a reduction of the complexity of the problem.

## 4 Conclusions

This paper reports on a numerical solution to the stochastic diffusive SFB model based on the calculation of Brownian dynamics trajectories associated with the Smoluchowski equation recently introduced by the authors.^19^ Given the large number of coordinates defining the SFB model, it is not convenient to solve numerically the Smoluchowski equation (e.g., by spanning the time evolution operator on a proper set of basis functions, leading to a linear algebra problem). Langevin equations are more tractable from a computational point of view (especially in terms of RAM memory occupation). The other side of the coin is that the BD trajectories must be long enough (and repeated enough times) to provide good statistics. This is in general not easy to ensure if one simply sets up a Brownian trajectory in Cartesian coordinates. The strength of the method presented here is that it is based on a modeling of the system that allows one to guide the setup of the BD trajectory calculations. Here, the global tumbling is sampled directly, as well as the effect of internal motions. Moreover, such a working paradigm allows one to perform a selection of which internal coordinates are relevant and which ones may be projected out in an attempt to reduce the system complexity (see below). The same procedure is not easily applicable when working with undifferentiated Cartesian coordinates. In the specific case of the SFB model, the time scales of motion are immediately accessed when transformed to the shifted **z** coordinates. Thanks to this information, it was easy to set up an automatic selection of the parameters of the BD trajectories, which makes the software package very easy to use. Finally, the implementation takes advantage of the GPU architecture, which is highly optimized for linear algebra problems with respect to CPU-based calculations. In particular, Cholesky decomposition is approximately linear with the dimension of the matrix (that is, with the number of atoms) until 10^4^. This is very important because the final objective is the application to proteins.

The harmonic approximation is not a real limit of the method. If the molecule is known (or a conformational analysis is performed in advance) to show multiple configurations of similar energy, then a simulation can be repeated in each relative minimum and then a Boltzmann weighting of the NMR relaxation data can be carried out. Such a procedure is strictly justified when the activation energy barrier from one minimum to another is high with respect to thermal energy. The Boltzmann weighting can be considered as a first rough approximation. However, soft activated coordinates can be included in the model by considering subsets of harmonic degrees of freedom linked by few activated modes: most of the algorithmic treatment is then left unchanged, with the introduction of jumping motions from a configuration to another. We plan to extend our model in this way to address partially structured proteins.

Provided that a good force field is available for the molecule under study, the approach has only one free parameter that regulates the results, i.e. the hydrodynamic radius or, more in general, the translational Stokes–Einstein friction of the atoms. Some testing is required to find a reference value of the hydrodynamic radius, but as a first observation, it should be said that the hydrodynamic radius should be smaller than the value commonly used in the DCM model (where no internal fast motions were included).

To optimize the application of the method, two routes can be explored. On the one hand, the employment of multiple time-step integration of the equations of motion can accelerate the simulation as part of the coordinates (the slowest ones) would be updated less frequently, in fact, reducing the complexity of the calculation. On the other hand, a Zwanzig projection of some of the internal fast coordinates can be carried out, based on the expected influence on the NMR relaxation data (this would also allow one to use a larger time step of integration). Both actions can be guided by analyzing the elements of the diffusion tensor **D**. The frequencies of the internal motions and the entity of coupling to the global rotational motion are important information to guide the eventual reduction in complexity. This is an important step for moving from small-medium molecules to macromolecules and active work is ongoing in our group in this direction.
